# The effects of terpenes on metabolism: a comprehensive review on recent updates

**DOI:** 10.1097/MCO.0000000000001129

**Published:** 2025-04-29

**Authors:** Efstathia Papada

**Affiliations:** Division of Medicine, University College London, London, UK

**Keywords:** diabetes, metabolism, plant-derived compounds, regulation, terpenes

## Abstract

**Purpose of review:**

There is a growing interest in plant-derived natural products as alternative means to manage chronic diseases. Terpenes represent a category of phytochemicals with several favourable effects including antioxidant and anti-inflammatory. The aim of this review is to explore the current understanding of how terpenes influence metabolism including glucose, lipid and amino acid and to discuss implications of these effects.

**Recent findings:**

Although most of the scientific evidence is derived from *in vitro* and animal studies, the effects of terpenes on metabolism have been also evaluated in limited human studies. In regard to the effects of terpenes on glucose metabolism they seem to interact with insulin signalling pathways, increase glucose uptake, inhibit enzymes and regulate the adenosine monophosphate-activated protein kinase (AMPK) pathway. Similarly, terpenes may contribute to modulation of key enzymes and genes involved in lipid metabolism. Their effects on amino acids metabolism are not well understood but it seems that some terpenes can modulate amino acid levels in the plasma, potentially influencing metabolic pathways related to protein synthesis, gene expression, and intracellular protein turnover.

**Summary:**

These effects can have significant implications for the management of chronic diseases such as diabetes and cardiovascular disease, as well as cancer, which is characterised by metabolic reprogramming. However, large-scale human studies are needed to conclude on terpenes effectiveness, safety and suitable dosage for favourable effects.

## INTRODUCTION

In recent decades, there has been a growing interest in discovering plant-derived natural compounds with biological properties as alternative means to manage various chronic diseases. Terpenes, a family of organic compounds primarily derived from plants, trees and citrus fruits have garnered significant attention. Apart from their ecological role in defending the plants themselves against herbivorous insects and pathogens, terpenes exhibit favourable effects on human health as they exhibit several biological properties, such as anti-inflammatory, antioxidant, anticancer, analgesic and antibacterial. They represent the largest and most diverse plant secondary metabolites in nature. The term ‘terpene’, introduced by Dumas in 1866, is derived from the Latin word for ‘turpentine’ (*Balsamum terebinthinae*), a resinous extract from pine trees [1].

The general chemical formula of terpenes (C_5_H_8_)_*n*_ is defined by the isoprene as a unit. They can be classified according to the number of isoprene units, the most common being monoterpenes (C_10_), sesquiterpenes (C_15_), diterpenes (C_20_), and triterpenes (C_30_). There are about 55 000 known terpenes and they can form terpenoids when functional groups as alcohols, aldehydes, or ketones appear in their chemical structure through processes like oxygenation, hydrogenation, or dehydrogenation. The chemical structure of monoterpenoids is based on two isoprene units (C_10_H_16_) with different arrangements as acyclic, monocyclic, and bicyclic. Sesquiterpenoids have three isoprene units (C_15_H_24_), with simple to complex mono- and polycyclic rings. Triterpenoids (C_30_H_48_) present with more than 40 different carbon skeletons. Tetraterpenoids (C_40_H_64_) have a high-molecular weight, and they are also known as carotenes [2].

There is a plethora of identified terpenoids which exhibit favourable metabolic effects, and as the list of terpenes increases annually, the research on terpenes effects on metabolism is attracting interest. This review explores the current understanding and recent findings on the mechanisms through which terpenes influence metabolism including glucose, lipid and amino acids and discusses implications of their metabolic regulation effects for health. 

**Box 1:**
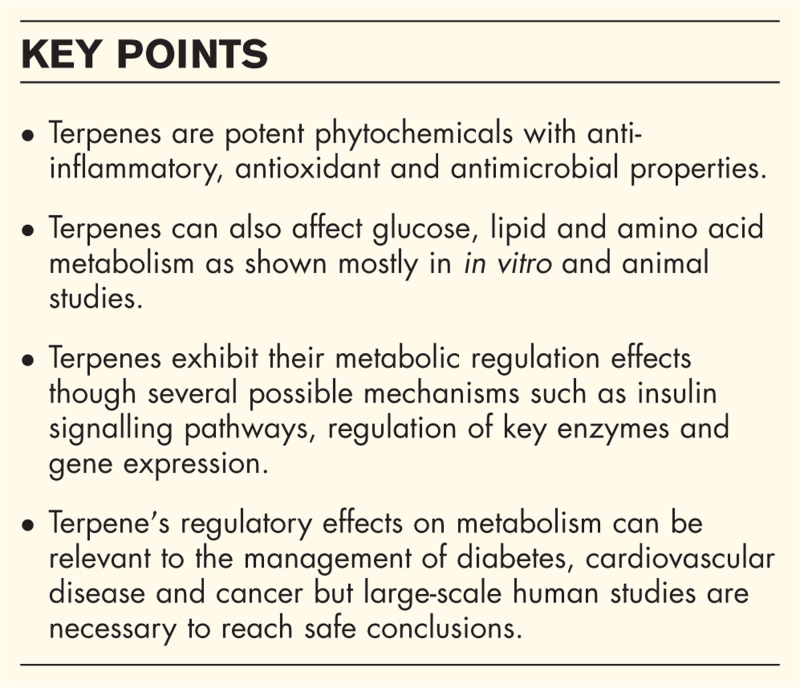
no caption available

## EFFECTS OF TERPENES ON METABOLISM

### Glucose metabolism

Terpenes seem to influence glucose metabolism, an effect which has been shown that could prove beneficial in the management of diabetes. Diabetes is a chronic metabolic disorder characterised by increased glucose concentration in the blood due to impaired insulin activity, sensitivity, or a combination of both. According to the International Diabetes Federation a total of 537 million adults aged 20–79 years are living with diabetes and this number is projected to rise to 643 million by 2030 [3]. Among other plant-derived compounds which have fewer side effects than conventional treatments, triterpenes, sesquiterpenes, and diterpenoids have shown a variety of pharmacological properties related to diabetes.

One of the key mechanisms by which terpenes influence glucose metabolism is through their interaction with insulin signalling pathways. Insulin resistance, a hallmark of type 2 diabetes (T2D), occurs when cells become less responsive to insulin, leading to elevated blood glucose levels. Terpenes have been shown to increase glucose uptake, glycogen synthesis, and insulin sensitivity by inhibiting the expression of α-glucosidase and α-amylase. By inhibiting these enzymes, terpenes can slow down the breakdown of carbohydrates into glucose, leading to lower postprandial blood glucose levels. They also inhibit the expression of plasma lipase, aldose reductase, tyrosine phosphatase, ghrelin, sodium/glucose cotransporter 1 (SGLT1), glucose transporter 2 (GLUT2), dipeptidyl peptidase-4 (DPP4), diacylglycerol acyltransferase (DGAT), hydroxysteroid dehydrogenase enzymes (HSD) and protein tyrosine phosphatase 1B (PTB-1B). Additionally, they regulate peroxisome proliferator-activated receptor gamma (PPAR-γ), incretins, inducible nitric oxide synthase (iNOS), and the adenosine monophosphate-activated protein kinase (AMPK) pathway [4].

**FIGURE 1:**
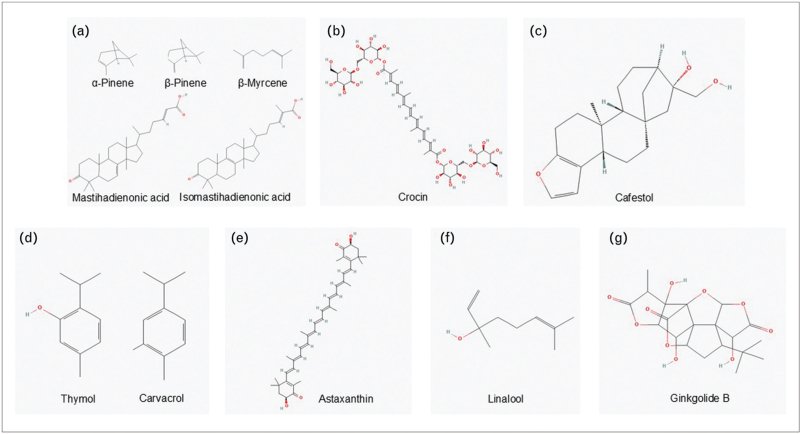
Examples of terpenes affecting glucose, lipid and amino acids metabolism. Mastiha's main terpenes: α-pinene, β-pinene, β-myrcene, mastihadienonic acid, isomastihadienonic acid, (b) crocin, (c) cafestol, (d) thymol, carvacrol, (e) astaxanthin, (f) linalool, (g) ginkgolide B (source of chemical structures: PubChem [31]).

Previous studies in humans have shown some promising results for terpenes in the management of blood glucose and insulin resistance. An example of these includes the triterpenes mastihadienonic (MNA) and isomastihadienonic acid (IMNA) (Fig. 1a) of the natural food product Mastiha (*Pistacia lentiscus*, var. *Chia*), which is a natural resin obtained from the Mastiha tree with several culinary uses in baking. Administration of Mastiha for 6 months induced significant reductions in insulin levels and insulin resistance in a double-blind, placebo-controlled, randomised trial on 21 healthy Japanese men over 40 years old, either when Mastiha was consumed alone or in combination with physical activity compared to a control group [5]. While there were no effects on fasting glucose, there were significant group by time statistical differences for insulin and HOMA-IR. Posthoc tests of insulin and HOMA-IR values adjusted for baseline showed that insulin and HOMA-IR were significantly reduced in Mastiha and physical activity group at 3 months and 6 months, and in Mastiha group at 3 months compared with the control. Another placebo-controlled, triple-blinded clinical trial studied the effects of crocin (Fig. 1b), a tetraterpenoid found mainly in saffron, and saffron in 150 patients with poorly managed T2D randomly divided into three groups (crocin, saffron and placebo) for three months. Comparison between groups showed that crocin and saffron led to a significant reduction in haemoglobin A1c (HbA1c) compared to placebo, and fasting blood glucose significantly reduced only in crocin compared to saffron and placebo groups [6].

Our most recent knowledge on the effects of terpenes on glucose metabolism though comes either from *in vitro* or animal studies. For example, a recent study investigated the effects of essential oils (EOs) derived from *Pinus* spp. (*P. nigra* and *P. radiata*) and their main terpenoid constituents, α- and β-pinene (Fig. 1a) on the expression/translocation of GLUT4 in myoblast C2C12 murine cells. After chemical profiling of the EOs through gas chromatography–mass spectrometry (GC–MS), cell viability was assessed by MTT assay, and GLUT4 expression/translocation was evaluated through RT-qPCR and flow cytometry analyses. The *P. nigra* essential oil (PnEO) and α-pinene increased the transcription of Glut4/Scl2a4 gene, resulting in an increase in GLUT4 production and its plasma membrane localisation. Additionally, the PnEO or α-pinene induced Glut4 expression both during myogenesis and in myotubes. Thus, the PnEO and α-pinene seem to mimic the effect of insulin on the GLUT4 transporter expression and its translocation to the muscle cell surface [7^▪^].

Cafestol (Fig. 1c), a bioactive terpenoid found in coffee, has shown glucose lowering and insulin secretory activity in cell and animal studies. In a randomised, double-blinded crossover acute intervention study, 15 healthy participants with increased waist circumference and thus elevated risk of developing T2D underwent three oral glucose tolerance tests one week apart, with placebo, 7 mg or 14 mg cafestol capsules ingested with the glucose load [8^▪^]. There were no differences in area under the curve (AUC) for glucose, insulin, glucagon-like peptide 1 (GLP-1) or gastric inhibitory peptide (GIP) with placebo or cafestol interventions. However, participants with impaired glucose tolerance and/or elevated fasting glucose, experienced a significant 11% larger AUC for GIP and a 5% nonsignificant smaller AUC for glucose, compared to placebo following ingestion of 14 mg cafestol pointing possibly towards better glucose regulation and higher insulin sensitivity. The same research team conducted a 12-week randomised, placebo-controlled, parallel trial in 40 healthy individuals with increased waist circumference at risk of developing T2D to assess the effects of purified cafestol on insulin sensitivity and other metabolic parameters [9^▪^]. Administration of 6 mg cafestol twice daily for 12 weeks did not change insulin sensitivity or glucose tolerance but induced significant reductions in body weight, visceral fat volume, and gamma-glutamyl transferase levels compared to placebo. Although no improvements on insulin sensitivity or glucose tolerance were observed cafestol might still contribute to the inverse association between coffee consumption and T2D.

Apart from their effects on glucose metabolism, it is important to mention that the anti-inflammatory and antioxidant properties of terpenes could be crucial in mitigating the chronic inflammation and oxidative stress associated with diabetes. Although terpenes exert multiple beneficial effects on glucose metabolism through various mechanisms, including enhancing insulin sensitivity and inhibiting carbohydrate-digesting enzymes further clinical studies are needed to fully elucidate these mechanisms.

### Lipid metabolism

As regards lipid metabolism terpenes seem to regulate lipid metabolism through several mechanisms including inhibition of hepatic lipid accumulation, regulation of steatosis, inhibition of hepatic lipogenesis, and promotion of fatty acid oxidation via modulation of key enzymes and genes [10].

An animal study on a hyperlipidaemic rat model evaluated the antioxidant and lipid-lowering effects of the essential oil of *Thymus satureioides* and its main compounds [11]. Terpenes thymol (26.6%) and carvacrol (25.9%) (Fig. 1d) were the most abundant volatile constituents, with a high vapor pressure and low water solubility, and the volatile oil displayed a lipid-lowering potential by reducing the levels of triglycerides, cholesterol, and nonhigh-density lipoprotein cholesterol (HDL-C) demonstrating a comparable antihyperlipidaemic effect to that of simvastatin.

Similarly, another monoterpene, α-pinene, has been shown to exert anti-inflammatory, hypoglycaemic, and hypolipidaemic properties in a rat model of diabetes decreasing cholesterol, triglyceride, very-low-density lipoprotein cholesterol (VLDL-C), low-density lipoprotein cholesterol (LDL-C), HDL-C [12^▪^]. It was assumed that similarly to a previous study with β-pinene by the same group [13] the mechanism of action includes stimulation of insulin secretion by blocking K^+^-adenosine triphosphate (ATP)-dependent channels in the membranes of pancreatic β-cells, causing depolarisation in the plasma membrane and consequently opening calcium channels and promoting the secretion of insulin vesicles into the bloodstream.

Astaxanthin (Fig. 1e), a terpene with anti-inflammatory and antioxidant properties, was evaluated in a randomised, double-blinded, placebo-controlled, crossover study with 15 male career firefighters [14]. Participants were supplemented with astaxanthin 12 mg/day or placebo for four weeks following a standardised resistance training program and performed a series of laboratory-based and occupation-specific tests to assess inflammatory markers, oxidative stress markers, cardiopulmonary exercise capacity, and performance to a fire ground test (FGT) consisting of nine fire suppressive activities. Astaxanthin improved occupation-related inflammation in response to exercise. Interestingly, while not statistically significantly different, fasting total cholesterol, triglycerides, LDL-C, non-HDL-C and VLDL-C were lower showing a more favourable lipid profile with astaxanthin treatment.

Another study evaluated the efficacy and safety of long-term use of a dietary supplement containing red yeast rice, combined with other natural compounds including astaxanthin, in children and adolescents with primary hypercholesterolaemia [15]. The supplement was administered once daily in 84 children/adolescents. Clinical and laboratory assessments were conducted before, 6 and 16 months after intervention. The supplement resulted in a significant decrease in total cholesterol, LDL-C, non HDL-C and apolipoprotein B levels, which was maintained with long-term administration. However, the results should be translated with caution as the supplement contained other phytochemicals as well which possibly act synergistically.

Furthermore, terpenes have been shown to influence lipid metabolism through their effects on gut microbiota as it has been reported that they can modulate gut microbiota composition and promote beneficial bacteria growth that can improve lipid metabolism. A study exploring the health effects of strawberry volatile organic compounds extract and its dominant terpene, linalool (Fig. 1f), showed a significant increase in the abundance of beneficial bacteria like *Lactobacillus, Bacillus* and *Alistipes* in mice [16^▪^]. Moreover, mice treated with linalool and strawberry extract demonstrated notable reductions in serum pro-inflammatory cytokines and triglyceride levels. *Bacillus* negatively correlated with glucolipid indices, and *Bifidobacterium* and *Dubosiella* negatively correlated with inflammatory factors, showing that changes in glucolipid metabolism might be associated with the regulation of gut microbiota and systemic inflammation.

Another study on an animal model of alcohol-related liver disease showed that a rich in triterpenes and flavonoids complex of *Pueraria lobata–Prunus mume* alleviated the disease by regulating lipid metabolism and inhibiting inflammation [17^▪^]. Administration of the complex reversed the increase in total cholesterol, triglycerides, LDL-C, normalised serum alanine transaminase (ALT), down-regulated Toll-like receptor 4- Nuclear factor kappa B (TLR4-NF-κB) signalling pathway to inhibit the release of tumour necrosis factor alpha (TNF-α), improved the expression of occludin, claudin-4 and tight junction protein ZO-1, and restored the abundance of *Muribaculaceae, Bacteroides* and *Streptococcus*.

These findings suggest that terpenes could be valuable in regulating lipid metabolism and related metabolic disorders. However, further clinical trials are needed to fully understand their efficacy and safety in humans.

### Amino acids

The effects of terpenes on amino acid metabolism are not fully understood and there is only limited evidence on their impact. Some terpenes have been shown to modulate the activity of enzymes involved in amino acid metabolism. For example, ginkgolide B (Fig. 1g), a terpene from *Ginkgo biloba*, seems to have a protective role against cerebral ischemic injury by inhibiting excitotoxicity and by modulating the imbalance of excitatory amino acids (such as glutamate and aspartate) vs. inhibitory amino acids (such as γ-aminobutyric acid (GABA) and glycine) [18]. This suggests that terpenes can influence the levels of specific amino acids in the brain, potentially affecting neurotransmitter balance and metabolic processes. More recent studies on humans provided insights into how terpenes influence the levels of plasma amino acids, which are crucial for numerous physiological functions.

An open label trial in 17 healthy humans investigated the impact of Mastiha's terpenes on plasma free amino acids [19]. Volunteers were instructed to follow a low-phytochemical diet for five consecutive days, that excluded fruits, vegetables, legumes, coffee, tea, alcoholic beverages and chocolate, aiming at minimising the concentration of circulating dietary phytochemicals. After overnight fasting participants were administered with Mastiha and blood samples were collected at different time points before and after Mastiha's ingestion to quantify the levels of 24 free amino acids using GC–MS. Branched-chain amino acids (BCAAs) such as valine decreased significantly four hours after ingestion, while proline and ornithine levels decreased at six and two hours respectively suggesting that terpenes can modulate amino acid levels in the plasma, perhaps by influencing metabolic pathways related to protein synthesis, gene expression, and intracellular protein turnover. Another randomised, double-blind, placebo-controlled trial in 60 patients with active inflammatory bowel disease (IBD) showed several changes in plasma amino acid levels following supplementation with Mastiha for 3 months as this ameliorated a decrease in plasma free amino acids seen in patients with ulcerative colitis taking placebo [20]. Similarly, in 68 patients with inactive IBD supplementation with Mastiha for 6 months inhibited an increase in plasma free amino acids seen in patients with quiescent IBD [21]. Change of amino acids is considered an early prognostic marker of disease activity and different circulating amino acids profiles have been reported in IBD reflecting nutritional state, but also inflammatory status and disease activity. For example, proline has been found upregulated in patients with IBD [22] and valine has been found significantly increased in DSS-treated mice [23]. All the above may indicate a potential role of Mastiha's terpenes in remission maintenance.

Further research is needed to fully elucidate the mechanisms underlying these effects and to determine the clinical relevance of terpenes in managing metabolic disorders related to amino acid metabolism.

## IMPLICATIONS OF THE REGULATORY EFFECT OF TERPENES ON METABOLISM

As discussed above the effects of terpenes on metabolism could prove effective in the management of chronic conditions such as diabetes and cardiovascular disease. Apart from these, their effects on regulation of metabolic pathways may be relevant also to other diseases, such nonalcoholic fatty liver disease (NAFLD) as shown in a randomised clinical trial with Mastiha's terpenes [24], and in metabolic dysfunction-associated steatotic liver disease (MASLD) as shown in a molecular docking study evaluating Mastiha's terpenoids as potential ligands for the glucocorticoid receptor (GR), liver X receptors (LXRα, LXRβ), PPARα, PPARγ, melanocortin 4 receptor (MC4R), AMPK and vascular endothelial growth factor receptor 2 (VEGFR2), whose up- and down-regulation interfere with MASLD development and progression [25]. Terpenes may also serve as potent candidates to the management of cancer, a disease characterised by metabolic reprogramming. Cancer cells exhibit uncontrolled proliferation, requiring substantial bioenergy and substances to synthesise essential biomolecules for growth and survival. Unlike normal cells, cancer cells adjust their metabolic processes including but not limited to glucose and lipid metabolism to support proliferation, balancing anabolic and catabolic needs to drive nutrients into biosynthesis and sustain their malignant state. Current first-line cancer treatments and drugs have limited success in altering metabolic reprogramming of cancer cells. Creating drugs that more effectively reprogram energy metabolism could improve the efficacy of antitumor therapies [26]. There is increasing evidence supporting the potential of targeted approaches in this area and terpenes may prove an effective therapeutic agent for cancer due to their metabolic effects. Accumulating evidence suggests that terpenes exert their effects through multiple molecular mechanisms, including regulation of apoptosis, induction of autophagy, inhibition of cellular signalling pathways, modulation of gene expression, inhibition of angiogenesis, and modulation of inflammation [27]. It has also been demonstrated that they can regulate the AMPK/peroxisome proliferator-activated receptor-gamma coactivator-1α (PGC-1α) signalling pathway [28]. This pathway is crucial in cancer cell metabolism by targeting energy homeostasis, mitochondrial biogenesis, glucose, and fatty acid oxidation, thus generating ATP for cell growth. Consequently, targeting this signalling pathway may offer a novel approach to cancer treatment and there are several preclinical studies *in vitro* and in animal models showing promising results for a plethora of terpenes such as ursolic acid, furanodiene, β-elemene, triptolide and oleanolic acid [28].

## CURRENT LIMITATIONS AND FUTURE RESEARCH NEEDS

Although terpenes could serve as a potent weapon in the management of several chronic diseases, there are several limitations and gaps in research to allow for their use in clinical practice. Even if they are bioavailable in humans [29,30], their bioavailability is low due to their hydrophobic nature and can limit their efficacy *in vivo.* Strategies such as encapsulation have been proposed to overcome this limitation, but further research in this area is needed. Additionally, there is a wide variability in sources of terpenes and their potential mechanisms, as well as in the appropriate dosages to ensure efficacy and safety. Thus, there is a need for standardisation of terpene preparations and further exploration of synergistic effects with other bioactive compounds. Most importantly, the majority of evidence comes from *in vitro* and animal studies thus there is a need for large-scale human studies to further explore the effects of terpenes on human metabolism.

## CONCLUSIVE REMARKS

In recent decades, terpenes, which are plant-derived compounds, have gained attention for their potential in managing chronic inflammation-mediated diseases due to their anti-inflammatory, antioxidant, anticancer, analgesic, and antibacterial properties. Interestingly, they also seem to affect metabolism by several proposed mechanisms including metabolic signalling pathways, enzymes activity regulation and gene expression among others. Due to these properties, terpenes have the potential to serve as an effective treatment for chronic diseases, such as diabetes and cardiovascular diseases, as well as cancer by regulating cancer metabolism. Further research on humans is needed so that bioavailability limitations, efficacy, dosage and safety parameters can be clarified.

## Acknowledgements

*None.*

### Financial support and sponsorship

*None.*

### Conflicts of interest

*There are no conflicts of interest.*

## REFERENCES AND RECOMMENDED READING

Papers of particular interest, published within the annual period of review, have been highlighted as:

▪ of special interest
▪▪ of outstanding interest
